# Inhibition of glycolysis and mitochondrial respiration promotes radiosensitisation of neuroblastoma and glioma cells

**DOI:** 10.1186/s40170-021-00258-5

**Published:** 2021-05-19

**Authors:** Donna L. Nile, Colin Rae, David J. Walker, Joe Canning Waddington, Isabel Vincent, Karl Burgess, Mark N. Gaze, Robert J. Mairs, Anthony J. Chalmers

**Affiliations:** 1 Institute of Cancer Sciences, University of Glasgow, Glasgow, G61 1QH UK; 2 Present Address: Integrated Covid Hub North East (ICHNE) Innovation Laboratory, Newcastle upon Tyne Hospitals NHS Foundation Trust, Newcastle upon Tyne, NE4 5BX UK; 3 Present Address: School of Medicine, University of Dundee, Dundee, DD1 4HN UK; 4 Glasgow Polyomics Facility, University of Glasgow, Glasgow, G61 1QH UK; 5 Present Address: Institute of Pharmacy and Biomedical Sciences, University of Strathclyde, Glasgow, G4 0RE UK; 6 Present Address: School of Biological Sciences, University of Edinburgh, Edinburgh, EH8 9XD UK; 7 Department of Oncology, University College London Hospitals NHS Foundation Trust, London, NW1 2BU UK

**Keywords:** Neuroblastoma, Radiation, Metabolism, 2-DG, Metformin, ^131^I-MIBG

## Abstract

**Background:**

Neuroblastoma accounts for 7% of paediatric malignancies but is responsible for 15% of all childhood cancer deaths. Despite rigorous treatment involving chemotherapy, surgery, radiotherapy and immunotherapy, the 5-year overall survival rate of high-risk disease remains < 40%, highlighting the need for improved therapy. Since neuroblastoma cells exhibit aberrant metabolism, we determined whether their sensitivity to radiotherapy could be enhanced by drugs affecting cancer cell metabolism.

**Methods:**

Using a panel of neuroblastoma and glioma cells, we determined the radiosensitising effects of inhibitors of glycolysis (2-DG) and mitochondrial function (metformin). Mechanisms underlying radiosensitisation were determined by metabolomic and bioenergetic profiling, flow cytometry and live cell imaging and by evaluating different treatment schedules.

**Results:**

The radiosensitising effects of 2-DG were greatly enhanced by combination with the antidiabetic biguanide, metformin. Metabolomic analysis and cellular bioenergetic profiling revealed this combination to elicit severe disruption of key glycolytic and mitochondrial metabolites, causing significant reductions in ATP generation and enhancing radiosensitivity. Combination treatment induced G_2_/M arrest that persisted for at least 24 h post-irradiation, promoting apoptotic cell death in a large proportion of cells.

**Conclusion:**

Our findings demonstrate that the radiosensitising effect of 2-DG was significantly enhanced by its combination with metformin. This clearly demonstrates that dual metabolic targeting has potential to improve clinical outcomes in children with high-risk neuroblastoma by overcoming radioresistance.

**Supplementary Information:**

The online version contains supplementary material available at 10.1186/s40170-021-00258-5.

## Background

Neuroblastoma comprises 7% of paediatric malignancies but has a worse prognosis than many other tumour types, accounting for approximately 15% of childhood cancer deaths [1]. Age at diagnosis, along with stage and molecular characteristics of the tumour, is used to allocate patients to low-intermediate- or high-risk groups. Younger age, low stage, and absence of segmental chromosomal abnormalities or oncogenic *MYCN* amplification are favourable features. However, most patients have high-risk disease by virtue of age greater than 12 months and distant metastatic disease or *MYCN* amplification [2]. The standard treatment schedule for high-risk neuroblastoma involves a complex multimodality schedule comprising induction chemotherapy [3], surgery [4], high-dose chemotherapy [5], radiotherapy [6] and immunotherapy [7]. While some patients respond well, some are refractory to initial treatments or relapse later. As neuroblastoma arises from primordial neural crest cells, most commonly in the adrenal medulla and also at other sites in the sympathetic nervous system, neuroblastoma cells usually retain the ability to take up catecholamines and analogues such as meta-iodobenzylguanidine (MIBG) [8]. Imaging with ^123^I-MIBG is used for staging and response assessment, and ^131^I-MIBG is a valuable treatment option [9].

The prognosis for patients with high-risk neuroblastoma has undoubtedly improved significantly over recent decades due to international cooperative group phase III trials investigating intensification of treatments such as high-dose chemotherapy and immunotherapy. Different sources yield different survival rates depending on the era of presentation, clinical trial selection factors, treatments used, and follow-up duration. Ten-year overall survival rates remain in the order of only 40% [10, 11], and while newer approaches offer the prospect of improvements in long-term survival, follow-up durations are currently insufficient to confirm these hopes [12]. Although ^131^I-MIBG is useful in the treatment of refractory and relapsed high-risk neuroblastoma and is the subject of clinical trials (ClinicalTrials.gov identifiers NCT02035137 and NCT03165292) aiming to optimise its use, further strategies are required to ensure that maximal benefit is obtained [13, 14]. It is likely that maximal therapeutic benefit from targeted radiotherapy will be obtained by its combination with drugs [14], which might render it more effective specifically for high-risk neuroblastoma patients, who do not benefit from existing therapies.

In recent years, a growing body of evidence has emerged demonstrating the anticancer properties of drugs that modulate cellular metabolism. Aberrant mitochondrial metabolism is a common trait among cancers. This is exemplified by the Warburg effect, which is characterised by increased rates of glycolysis and lactate production, even in the presence of oxygen [15]. Dysregulated cancer cell metabolism has been linked to increased tumour aggressiveness and treatment resistance [16]. Indeed, targeting glucose metabolism has yielded chemo- and radiosensitisation in several cancer types [17, 18]. Of relevance to this study, neuroblastoma cells have been reported to exhibit aberrant metabolic properties, including increased fatty acid synthase expression [19] and altered fatty acid and glucose metabolism [20].

The synthetic glucose analogue 2-deoxyglucose (2-DG) is avidly taken up by rapidly proliferating cells, including cancer cells. Once internalised, 2-DG is unable to undergo isomerisation at its second carbon atom to produce 2-deoxyglucose-6-phosphate, a phenomenon that leads to inhibition of glycolysis at the phosphoglucoisomerase level. Therefore, treatment with 2-DG becomes cytotoxic in the contexts of ATP depletion [21] or disruption of glutathione metabolism [22]. In keeping with this, 2-DG was shown to be an effective radiosensitiser of HeLa cells [22] and was well tolerated in combination with docetaxel in a phase 1, dose-escalation clinical trial [23].

Metformin is a biguanide drug that is widely prescribed for the treatment of type 2 diabetes, acting to reduce insulin resistance, enhance glucose utilisation and reduce hepatic glucose production [24]. At the cellular level, metformin promotes AMPK activation during energy homeostasis via inhibition of Complex I of the mitochondrial respiratory chain [25, 26]. Indeed, metformin has been shown to exhibit antineoplastic activity in the context of exogenously induced changes in energy homeostasis [27]. Additionally, patients with type 2 diabetes who were receiving metformin as part of their diabetic management have been shown to experience reduced incidence and better survival rates for many solid cancers [28–30].

In keeping with these observations, both 2-DG and metformin have shown potential as radio- [22, 31–34] and chemosensitisers [23, 35, 36]. However, their ability to sensitise neuroblastoma cells to radiotherapy has not yet been studied, and the mechanisms underlying their radiosensitising properties are poorly understood. Accordingly, we investigated the potential of 2-DG and metformin to enhance the efficacy of X-irradiation in pre-clinical models of neuroblastoma. We show that 2-DG effectively radiosensitises neuroblastoma and glioma cells, effects that are significantly enhanced in the presence of metformin, and demonstrate that combining 2-DG, metformin and radiation induces extensive metabolic reprogramming. This is characterised by reduced cellular respiration and ATP production that associates with prolonged G_2_/M arrest during which cells ultimately succumb to apoptotic cell death. Our results indicate that dual pharmacological inhibition of glycolysis and mitochondrial respiration has potential to enhance the efficacy of radiation therapy in patients with neuroblastoma.

## Materials and methods

### Reagents

2-Deoxy-D-glucose (2DG; D3179) and metformin hydrochloride (PHR1084) were purchased from Sigma-Aldrich (Poole, UK) and were reconstituted using sterile water. Drugs were then diluted in culture medium. Unless otherwise stated, all other cell culture reagents were purchased from Thermo Fisher Scientific (Paisley, UK) and all chemicals were purchased from Sigma-Aldrich (Poole, UK).

### Cell culture

Human neuroblastoma SK-N-BE(2c) cells were purchased from the American Type Culture Collection and were maintained in Dulbecco’s modified Eagle medium (DMEM) containing 15% (v/v) fetal calf serum, 2 mM L-glutamine and 1% (v/v) non-essential amino acids. Human glioblastoma UVW cells were transfected with a plasmid containing the bovine noradrenaline transporter (NAT) gene [37] and were maintained in minimum essential medium (MEM) containing 10% (v/v) fetal calf serum, 2 mM L-glutamine, 1% (v/v) non-essential amino acids and 1 mg/ml geneticin. Human neuroblastoma CHLA-20 cells were obtained from the Children’s Oncology Group Childhood Cancer Repository (TX, USA) and were maintained in Iscove’s modified Dulbecco’s medium (IMDM) containing 20% (v/v) fetal calf serum, 2 mM L-glutamine and 1% (v/v) insulin-transferrin-selenium. All cells were incubated at 37 °C, 5% CO_2_ in a humidified incubator, and were passaged every 3–4 days. Cell lines were cultured in this study for less than 6 months after resuscitation and were deemed free of Mycoplasma contamination by in-house testing using the MycoAlert mycoplasma detection kit (Lonza, Bazel, Switzerland).

### MTT cell growth assay

The MTT reduction assay was used to estimate the effect of drug treatment on cell growth up to 96 h post treatment. After seeding cells in 96-well plates, the following day, cells were washed with PBS before adding 200 μl drug-containing medium. After 24 h, 48 h, 72 h, and 96 h, cells were incubated for 2 h with 20 μl MTT solution (5 mg/ml MTT in PBS). Internalised MTT was solubilised with DMSO and absorbance was measured at 570 nm.

### Clonogenic assay

Monolayers were cultured in 25 cm^2^ flasks before 24-h drug treatment and simultaneous irradiation (Xstrahl RS225 X-Ray irradiator, Xstrahl Limited, Surrey, UK; dose rate of 0.93 Gy/min) at the stated concentrations and doses. Irradiation was always administered at the beginning of drug treatment, with the only exception being during the alternative scheduling experiments (as further detailed in the text). Clonogenic survival was determined by seeding 500 (SK-N-BE(2c)) or 250 (UVW/NAT) cells in to 21.5 cm^2^ petri dishes as previously described [38].

### Spheroid growth delay

Cells were cultured in 25 cm^2^ flasks coated with 1% (w/v) agar to initiate multicellular spheroid formation. After 3 days, spheroids were transferred to sterile universal tubes before treatment in serum-free drug-containing medium and simultaneous irradiation. Spheroids were treated for 24 h, and those of approximately 100 μm in diameter were individually transferred to agar-coated 24-well plates, with 12 replicate spheroids per condition, as previously described [39, 40]. Each spheroid was imaged every 3–4 days for 3 weeks using an inverted phase contrast microscope. The longest spheroid length, *d*_max_, and its perpendicular width, *d*_min_, were measured using ImageJ (NIH, Bethesda, MD). Tumour volume, *V* (μm^3^) was then estimated assuming *V* = *π* × *d*_max_ × *d*_min_^2^/6 [41], which allowed determination of the area under the *V*/*V*_0_ versus time curve (AUC) using trapezoidal approximation [39, 40].

### Untargeted metabolomic analysis

Hydrophilic interaction liquid chromatography (HILIC) was carried out on a Dionex UltiMate 3000 RSLC system (Thermo Fisher Scientific, Hemel Hempstead, UK) using a ZIC-pHILIC column (150 mm × 4.6 mm, 5-μm column, Merck Sequant). The column was maintained at 30 °C and samples were eluted with a linear gradient (20 mM ammonium carbonate in water and acetonitrile) over 26 min at a flow rate of 0.3 ml/min. The injection volume was 10 μl and samples were maintained at 5 °C prior to injection. Metabolites were detected using the Thermo Orbitrap Q Exactive (Thermo Fisher Scientific) mass spectrometer at a resolution of 70,000 (at 70–1050 m/z), which was operated in polarity switching mode. A pooled sample comprising a mixture of all sample extracts was analysed using the same conditions and was run every 5th sample. Instrument .raw files were converted to positive and negative ionisation mode mzXML files. These files were then analysed using the XCMS [42]/MZMatch [43]/IDEOM pipeline. Polyomics integrated Metabolomics Pipeline (PiMP) and FrAnK were used to assess fragmentation data [44]. The weighted distance of treatment conditions to untreated control samples was calculated using the following equation:

$$ \mathrm{Weighted}\ \mathrm{distance}=\sqrt{\left(\Big( PC1\ {rep}_n- PC1\ {Ctrl}_{mean}\right)\times 0.49\Big){}^2+{\left(\left( PC2\ {rep}_n- PC1\ {Ctrl}_{mean}\right)\times 0.218\right)}^2\ } $$

where PC1rep_n_ is the PC1 value of each replicate and PC1Ctrl_mean_ is the mean PC1 value of all untreated control samples [45]. All metabolomic data has been uploaded to the repository MetaboLights (https://www.ebi.ac.uk/metabolights/).

### Bioenergetic analysis of glycolysis and mitochondrial respiration

UVW/NAT cells and CHLA-20 cells were seeded at a density of 1 × 10^4^ and 3 × 10^4^ cells, respectively, in a volume of 175 μl in Seahorse XFe96-well plates (Cat# 101085-004, Seahorse Biosciences, Agilent Technologies, Cheshire, UK). Cells were allowed to settle for 2 h at room temperature, before drug treatment (25 μl) and simultaneous irradiation, as required. Mitochondrial respiration was measured 24 h after treatment using the Seahorse XFe96 Analyzer (Seahorse Biosciences, Agilent Technologies, Cheshire, UK) according to manufacturer’s instructions. On the day of the assay, culture medium was replaced with Seahorse XF base medium (Cat# 102353-100), supplemented with the regents required for each cell line (except FBS), as detailed above. Cells were then incubated for 1 h at 37 °C in the absence of CO_2_. Using the Seahorse Cell Mito Stress Test (Cat# 103015-100), sequential injections were made of oligomycin (1 μM), carbonyl cyanide-p-trifluoromethoxy-phenylhydrazone (FCCP) (1 μM), rotenone (0.5 μM) and antimycin A (0.5 μM) to test cellular response to mitochondrial stress in live cells. Mitochondrial oxygen consumption rate (OCR) and extracellular acidification rate (ECAR) were measured every 3 min, for a total of 75 min. The OCR and ECAR traces were then used to estimate basal and maximal respiration, ATP synthesis, and glycolytic rate as described previously [46]. Data were normalised to protein content, which was determined after each Seahorse assay using the Pierce Micro BCA Protein Assay kit (Cat# 23235, Thermo Fisher Scientific, Paisley, UK), following manufacturer’s instructions.

### Fluorescence-activated cell sorting (FACS) analysis

Cells were seeded at a density of 7 × 10^5^ (SK-N-BE(2c)) or 4 × 10^5^ (UVW/NAT) cells in 75 cm^2^ flasks. Drug-containing medium was added once cells were 60% confluent, and cells were simultaneously irradiated. After 24 h, cells were trypsinised and washed with PBS, before fixing with 70% (v/v) ethanol in water at − 20°C for a minimum of 6 h. Ethanol was removed by washing with PBS and cells were resuspended in PBS containing propidium iodide (10 μg/ml) and RNase A (200 μg/ml), before analysis using a BD FACSVerse flow cytometer (BD BioSciences, Oxford, UK). FACS data were quantified using FlowJo 7.6.5 software. Our gating strategy is provided in Supplementary Information Fig. S8a.

### Live cell imaging

For real-time analysis of cellular proliferation and apoptotic frequency, UVW/NAT and CHLA20 cells were seeded in 96-well plates at densities of 1.5 × 10^3^ or 1.75 × 10^4^ cells/well, respectively. After overnight incubation at 37 °C, Annexin V-labelled cells (IncuCyte Annexin V Red, Cat# 4641, Essen BioScience, 1:400) were analysed with the IncuCyte ZOOM live cell imaging system (Essen BioScience, Hertfordshire, UK) following drug treatment (as detailed in the text). Each condition was replicated in six wells per experiment for each cell line. Two images per well were acquired every hour at a magnification of × 10, over a time period of 50 h. The number of Annexin V-positive cells was normalised to total cell confluence, using the IncuCyte ZOOM 2018A software (Ann Arbor, MI, USA).

## Results

### Radiosensitising effect of 2DG and metformin as single agents

**Fig. 1:**
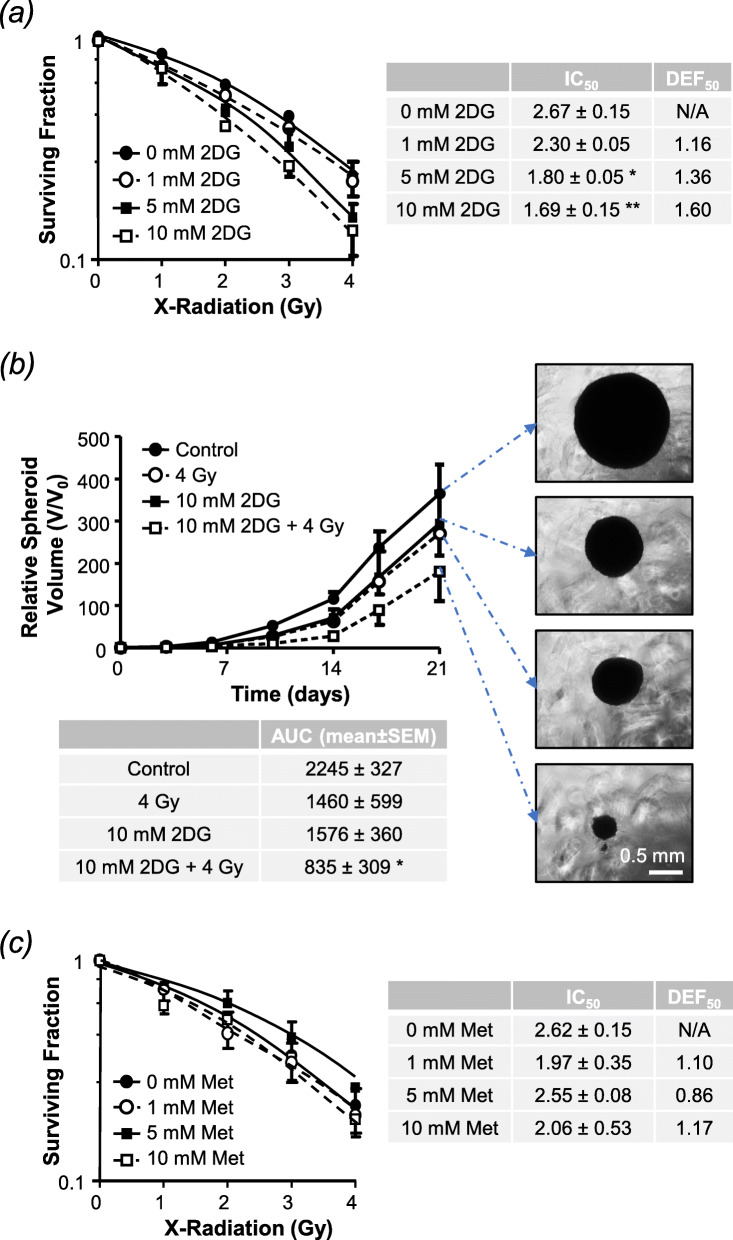
Radiosensitising effect of 2DG and metformin as single agents. The clonogenic survival of UVW/NAT cells following 24-h treatment with **a** 1, 5, or 10 mM 2DG or **c** 1, 5, or 10 mM metformin in the presence 1–4 Gy X-radiation (administered at the beginning of drug treatment). The 50% inhibitory concentration (IC_50_) and the dose enhancement factor observed at the 50% kill level (DEF_50_) are shown. **b** Growth inhibition of SK-N-BE(2c)-derived multicellular spheroids (*n* = 3) after 24-h treatment with 4 Gy X-radiation or 10 mM 2DG, as single agents or in combination. Data expressed as percentage are under the volume-time curve (AUC) with representative spheroids shown after 21-day treatment, × 5 magnification. *p < 0.05, **p < 0.01 compared to untreated control cells. All data are mean ± SEM from 3 experimental repeats

Both 2-DG and metformin exhibited modest activity as single agents, with reductions in cell proliferation and clonogenic survival being observed after exposure to concentrations greater than 1 mM 2DG or 10 mM metformin (Supplementary Information Fig. S1a-d). When delivered for 24 h commencing at the time of external beam radiation, 2-DG reduced the clonogenic capacity of UVW/NAT cells in a dose-dependent manner, with statistically significant radiosensitisation observed at 5 mM and 10 mM, with dose enhancement factors at 50% cell kill (DER_50_) of 1.80 and 1.69, respectively (Fig. 1a). Similarly, 10 mM 2-DG significantly enhanced X-irradiation induced growth delay in SK-N-BE(2c)-derived spheroids, with the combined effect being greater than either agent administered alone (Fig. 1b). In contrast, metformin had negligible effect on clonogenic survival of UVW/NAT cells when administered in combination with irradiation (Fig. 1c), results that were confirmed in SK-N-BE(2c) cells (Supplementary Information Fig. S2).

### Combined effects of 2-DG and metformin on radiosensitisation

The failure of metformin to radiosensitise neuroblastoma and glioma cells contrasts with data from previous studies on pancreatic [31] and breast cancer cells [34]. However, there is a rationale for combining metformin treatment with 2-DG treatment. Inhibition of glycolysis by 2-DG is predicted to cause a compensatory increase in mitochondrial respiration to maintain ATP production. Combination treatment with metformin would circumvent this compensatory increase in mitochondrial metabolism by inhibiting Complex 1 of the respiratory chain. There is also evidence of supra-additive activity of 2-DG and metformin combination treatment in other cancer types [47–49]. Most pertinently, Chatterjee and colleagues demonstrated that simultaneous administration of 2-DG and metformin enhanced radiation-induced cytotoxicity in two breast cancer cell lines [50].

**Fig. 2:**
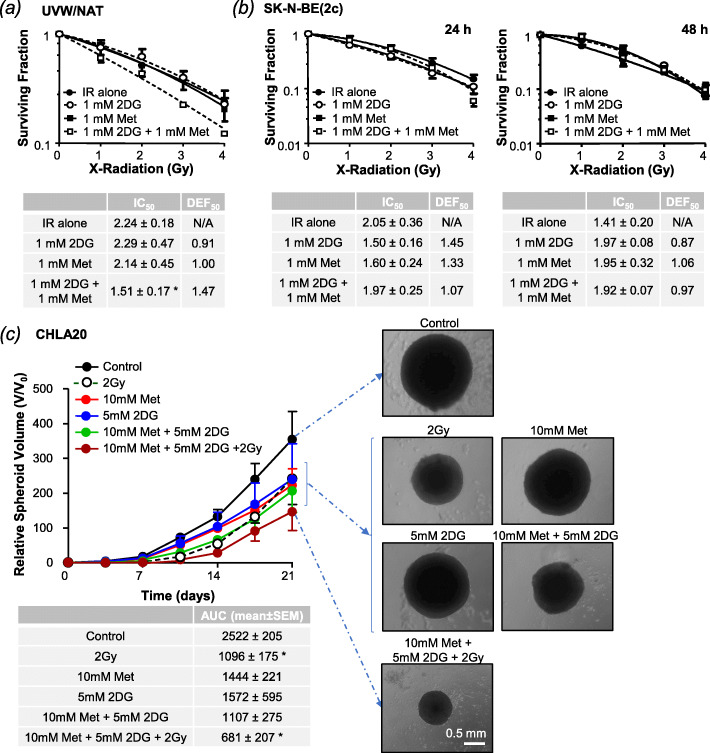
Combined effect of 2DG and metformin on radiosensitisation. The effect of 2DG and metformin on the radiation-induced cell kill of **a** UVW/NAT (*n* = 3) cells after 24h drug treatment and **b** SK-N-BE(2c) cells after 24h (*n* = 4) or 48h (*n* = 2) drug treatment. **c** Growth inhibition of CHLA20-derived multicellular spheroids (*n* = 3) after 24h treatment with 2 Gy X-radiation (at the beginning of treatment), 10 mM metformin, or 5 mM 2DG, as single agents or in combination. Data expressed as percentage are under the volume-time curve (AUC). Representative CHLA20-derived spheroids are shown after 21-day treatment, × 5 magnification. Data are means ± SEM from the stated number of experimental repeats. *p < 0.05, **p < 0.01, ***p < 0.001 compared to irradiated (IR) cells (**a**, **b**) or untreated control cells (**c**)

In keeping with this rationale, 2-DG and metformin exerted additive effects on clonogenic cell kill in the absence of radiation (Supplementary Information Fig. S1e-f). Likewise, dual treatment with low doses of 2-DG and metformin (1 mM) significantly radiosensitised UVW/NAT cells (drugs administered for 24 h commencing at the time of irradiation, Fig. 2a) but failed to radiosensitise SK-N-BE(2c) cells (Fig. 2b).

Since SK-N-BE(2c) cells exhibit a longer population doubling time (37 h) than UVW/NAT cells (16 h), we postulated that more prolonged drug treatment might be required to radiosensitise these cells. However, 48h exposure to metformin and 2-DG also failed to increase radiation-induced cell kill (Fig. 2b).

Because of the discrepancy between UVW/NAT glioma cells and SK-N-BE(2c) neuroblastoma cells, we evaluated an alternative human neuroblastoma cell line, CHLA-20. These cells were unable to form the colonies required for clonogenic assay but readily formed three-dimensional spheroids. In this clinically relevant model, we observed enhanced spheroid growth delay following triple combination therapy, as opposed to single agent modalities (Fig. 2c).

### Combination treatment significantly disrupts glycolysis, mitochondrial respiration and nucleotide metabolism

**Fig. 3:**
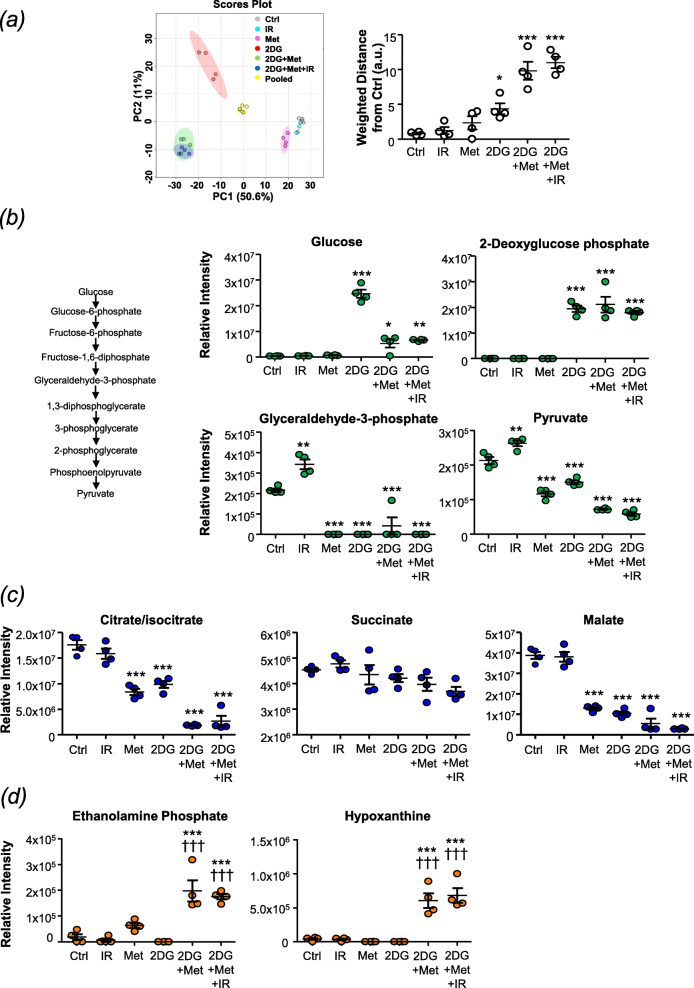
Effect of combination treatment on glycolytic and mitochondrial metabolites. UVW/NAT cells were treated with 3 Gy X-radiation, 1 mM metformin, or 2.5 mM 2DG as single agents or in triple combination for 24 h. Cells were then harvested in solvent extraction buffer and analysed by mass spectrometry in quadruplicate. **a** Principal component analysis (PCA) plot showing biological variation within the data set, which was then used to calculate the weighted distance between each culture condition and their respective mean values ± SD. Intracellular relative intensity of **b** glycolytic intermediates (including a schematic representation of the glycolytic pathway), **c** tricarboxylic acid (TCA) cycle metabolites and **d** by-products of purine and pyrimidine metabolism. Data are means ± SD, *n* = 4. Treatment vs control: *p < 0.05, **p < 0.01, ***p < 0.001; treatment vs 2-DG: ^†††^*p* < 0.0001 following a one-way ANOVA with Bonferroni correction

To understand why metformin enhanced the radiosensitising potential of 2-DG in UVW/NAT cells, we interrogated the effect of single agent and combination treatments on glycolytic and mitochondrial respiration metabolites. An initial untargeted analysis using a principal component plot revealed that 2-DG induced significant changes in the metabolomic composition of UVW/NAT cells, whether administered as a single agent or in combination (Fig. 3a). Further interrogation of glycolytic pathway metabolites revealed 2-deoxyglucose phosphate to be significantly increased in cells exposed to 2-DG treatment, proving that 2-DG was internalised and phosphorylated by hexokinase (Fig. 3b). This phosphorylation rendered 2-DG refractory to metabolism by glycolysis, as shown by the reduction in glyceradehyde-3-phosphate, phosphoenolpyruvate and pyruvate, the end-product of glycolysis, in all treatments involving 2-DG (Fig. 3b and Supplementary Information Fig. S3a).

Likewise, investigation of the tricarboxylic acid (TCA) cycle (Supplementary Information Fig. S3c) revealed citrate/isocitrate and malate to be significantly reduced following exposure to single agent or combination therapy involving 2-DG (Fig. 3c). Single agent metformin treatment also significantly reduced the relative abundancies of these metabolites, demonstrating inhibition of Complex 1 of the respiratory chain.

Our metabolomic analysis also revealed that 2-DG single agent treatment promoted large-scale changes in purine and pyrimidine metabolism. Specifically, we observed a decreased abundance in 20 of 26 metabolites detectable within the purine metabolic pathway (Supplementary Information Fig. S4). Similarly, 2-DG promoted a reduction in 11 of the 27 metabolites we detected within the pyrimidine metabolic pathway (Supplementary Information Fig. S5). Comparable results were observed after metformin single agent therapy, but to a lesser extent (data not shown). Further analysis of combination treatments revealed that dual 2-DG/metformin and triple 2-DG/metformin/radiation combination therapies significantly increased the abundance of ethanolamine phosphate and hypoxanthine (Fig. 3d; *p* < 0.001), which are products of serine and adenine metabolism, respectively. Interestingly, the increased abundance of these two metabolites was only observed following combination treatments.

### Metabolite imbalance following therapeutic intervention culminates in impaired cellular respiration

Having demonstrated marked disruption of glycolysis and mitochondrial metabolism by our combination drug treatment, we explored its effects on cellular respiration and energy production. The mitochondrial respiratory profile of UVW/NAT and CHLA-20 cells was analysed using the Seahorse XF system, whereby live cells were sequentially injected with different test compounds to induce mitochondrial stress (Supplementary Fig. S6).

**Fig. 4:**
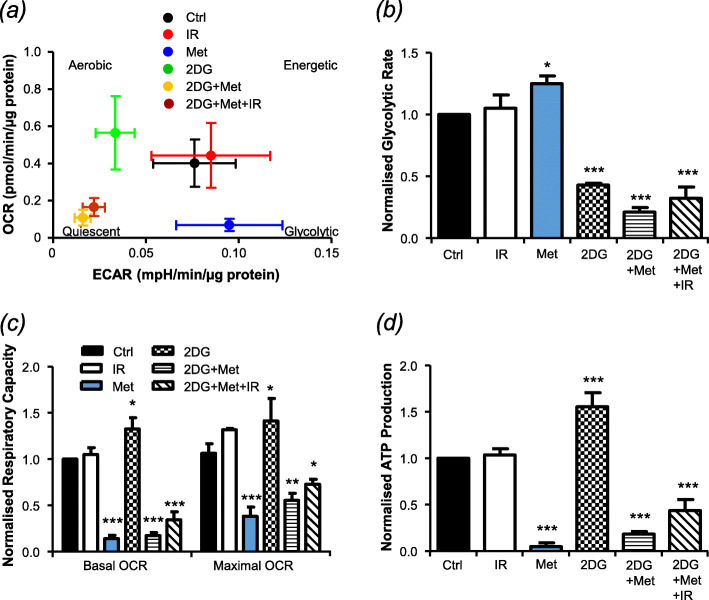
The effect of combination treatment on mitochondrial respiration, glycolysis and cellular energy production. UVW/NAT cells were treated with 3 Gy X-radiation, 1 mM metformin, or 5 mM 2DG as single agents or in combination for 24 h. Oxygen consumption rate (OCR) and extracellular acidification rate (ECAR) of live cells were then measured using a Seahorse XFe96 Analyser, the shift in cell energy phenotype following treatment is shown (**a**) The traces obtained (Supplementary Figure 6) were then used to calculate glycolytic rate (**b**), minimal and maximal respiratory capacity (**c**) and ATP production (**d**)

Initially, basal extracellular acidification rate (ECAR) was plotted against mitochondrial oxygen consumption rate (OCR) (Fig. 4a). While these bioenergetic profiles were unaffected by irradiation (3 Gy), 2-DG treatment promoted aerobic respiration, presumably in response to glycolytic inhibition, whereas metformin-treated cells became increasingly glycolytic as mitochondrial respiration was inhibited (Fig. 4a, b). The greatest shift in bioenergetics was seen following combination therapy involving 2-DG and metformin in the presence or absence of radiation, with cells becoming less energetic and more quiescent as the two central ATP production pathways were inhibited. Interestingly, single agent metformin produced the greatest shift in mitochondrial respiratory capacity, with no additional effect observed when 2-DG or radiation were added (Fig. 4c). Single agent 2-DG treatment, however, significantly increased both the respiratory capacity and ATP production of UVW/NAT cells. Our hypothesis that this reflected increased utilisation of more efficient mitochondrial ATP production was supported by the reversal of the effect that was observed when metformin was added (with or without irradiation, Fig. 4c, d). Comparably, combination treatment significantly reduced abundance of the metabolites AMP, ADP and ATP (Supplementary Information Fig. S3d). Similar results were obtained in CHLA-20 cells (Supplementary Fig. 7).

Taken together, these results demonstrate that 2-DG and metformin treatment caused significant disruption of glycolysis and/or the mitochondrial TCA cycle, which culminated in significantly impaired cellular energy production. However, this effect was not enhanced in the presence of irradiation, even though both drugs were required to enhance radiosensitivity.

### Triple combination therapy significantly prolongs G_2_/M cell cycle arrest

**Fig. 5:**
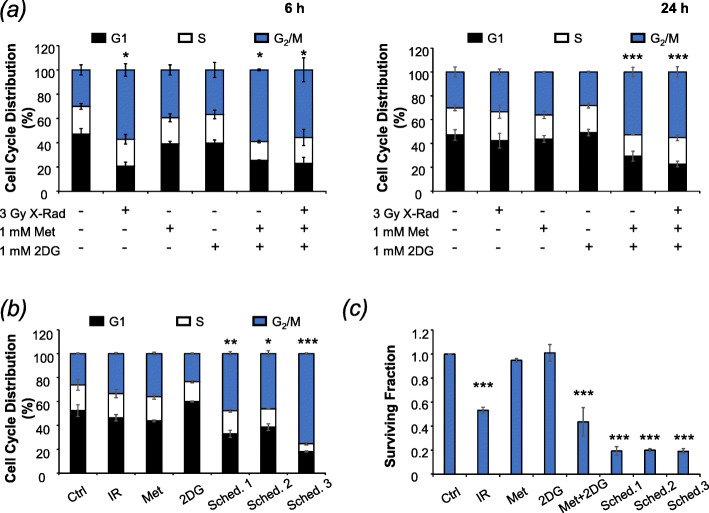
The effect of 2DG and metformin on the cell cycle and alternative scheduling. **a** UVW/NAT cells were treated with 3 Gy X-radiation, 1 mM metformin, or 1 mM 2-DG, as single agents or in simultaneous combination for 6 h or 24 h, and cell cycle distribution was determined following propidium iodide staining and flow cytometric analysis. The effect of scheduling on **b** the cell cycle and **c** clonogenic survival (*n* = 4). Data presented are means ± SEM from 3 independent experiments, unless otherwise stated. *p < 0.05, **p < 0.01, ***p < 0.001 compared to untreated control cells

To further understand the mechanisms underlying the efficacy of triple combination therapy, effects on cell cycle progression were investigated using the gating strategy presented in Supplementary Fig. S8a. Single agent 2-DG treatment at doses of 2.5 mM and above caused accumulation of cells in G_2_/M phase of the cell cycle after 6 h, whereas metformin had negligible effect on the proportion of G2/M phase cells at any dose (Supplementary Fig. S8b-c). However, combined low-dose (1 mM) treatment with 2-DG and metformin caused a marked G_2_/M arrest that was observed 6 h after treatment and failed to resolve at 24 h (Fig. 5a). Radiation alone (3 Gy) caused a temporary G2/M arrest, as expected, but did not amplify cell cycle effects of the drug combination (Fig. 5a).

Our previous studies have demonstrated the importance of scheduling when combining treatment modalities [39, 51], and we predicted that accumulation of cells in G_2_/M phase of the cell cycle would increase radiosensitivity [52] by doubling the amount of DNA susceptible to radiation damage in each cell. To investigate this possibility, three different schedules were tested: (1) three treatments simultaneously, (2) 2-DG and metformin 6 h prior to irradiation, and (3) irradiation 6 h prior to 2-DG and metformin (Supplementary Fig. S8d). Cells were harvested after a total treatment time of 24 h and analysed by propidium iodide staining and flow cytometry (Fig. 5b) or clonogenic assay (Fig. 5c), to determine effects on cell cycle distribution and cell survival, respectively. The 6h interval was selected because maximum G_2_/M arrest was observed at this timepoint (Fig. 5a) [38].

Maximum G_2_/M arrest at 24 h was observed when cells were irradiated 6 h prior to drug therapy (Schedule 3, Fig. 5b). Schedules 1 and 2 were less effective, yielding 2-fold increases in the proportion of cells in G_2_/M phase. While all three combination schedules enhanced clonogenic cell kill compared to individual treatments (Fig. 5c), no difference in efficacy was observed between the different schedules. These results demonstrate that increased G_2_/M arrest does not necessarily translate into increased clonogenic cell kill for this combination.

### Sustained G_2_/M arrest promotes apoptotic cell death

**Fig. 6:**
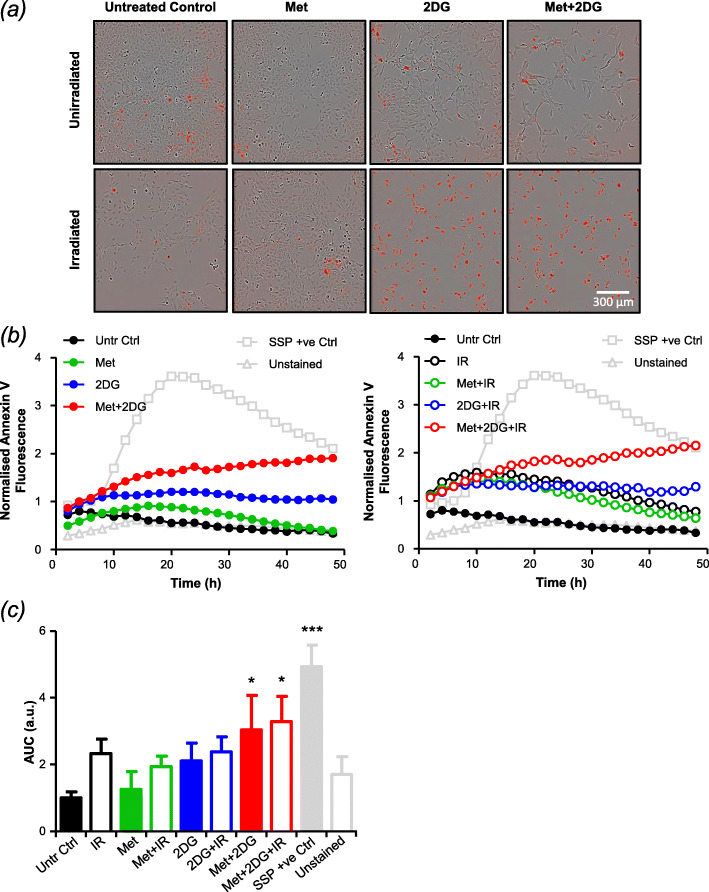
The effect of combination treatment on apoptotic frequency. UVW/NAT cells were treated with 2 Gy X-radiation (administered at the start of treatment), 1 mM metformin, or 1 mM 2DG as single agents or in combination for 48 h and were imaged every hour using the IncuCyte Zoom imaging system. Representative images taken after 48h treatment exposure are shown (**a**). Apoptotic frequency was determined following Annexin V staining. Annexin V-positive cells (displaying red fluorescence) were normalised to the total number of cells present (**b**) and the area under the curve (AUC) was calculated (**c**) to allow comparisons between each condition and the treatment controls (untreated control (Untr Ctrl), unstained negative control (unstained) and staurosporine positive control (SSP +ve)). Data are means ± SEM from 3 separate experiments, each performed in sextuplet wells. *p < 0.05, **p < 0.01 compared to untreated control cells (one-way ANOVA)

To interrogate the possibility that prolonged G_2_/M arrest following triple combination therapy would culminate in apoptotic cell death, we used IncuCyte live cell imaging to determine the proportion of cells expressing the apoptotic marker Annexin V at multiple timepoints after treatments. These data showed that 2-DG but not metformin caused modest levels of apoptotic cell death as a single agent, with dual drug therapy showing a supra-additive effect in UVW/NAT cells (Fig. 6a, c). Radiation alone induced moderate apoptosis at early timepoints but did not further enhance efficacy of the 2-DG/metformin combination (Fig. 6b, c). Similar results were observed using CHLA-20 cells (Supplementary Fig. S9).

## Discussion

Despite rigorous multi-modal treatment regimens involving surgical resection, chemotherapy, radiotherapy and immunotherapy, high-risk neuroblastoma patients have 10-year overall survival rates of less than 40% [10, 11]. Targeted radiotherapy remains one of the most effective treatment options for high-risk disease [53], but most patients are not cured and so there is scope to improve its efficacy by using targeted drugs to overcome treatment resistance [14].

In this study we aimed to enhance radiosensitivity by inhibiting glycolysis, which is upregulated in cancer cells, thus disrupting the ATP generation required to fuel their rapid proliferation. Anticipating a compensatory increase in mitochondrial metabolism to maintain ATP production, we hypothesised that this could be circumvented by adding the mitochondrial respiratory chain inhibitor metformin. Building on recent observations in breast cancer cells [50], we tested these hypotheses by measuring the effects of 2-DG and metformin on the radiation responses of neuroblastoma and glioma cells.

Metformin failed to radiosensitise neuroblastoma (SK-N-BE(2c)) or glioma (UVW/NAT) cells when administered as a single agent, which is contrary to previous reports [31, 32, 34]. However, we did observe that low-dose 2-DG and metformin given in combination significantly increased the radiosensitivity of UVW/NAT cells but had negligible effects on SK-N-BE(2c) neuroblastoma cells, even when treatment was extended to 48 h. These observations are consistent with previous reports demonstrating a range of sensitivities to 2-DG and metformin combination treatment across cell lines [47–49]. Variability in response to metformin has been attributed to genetic characteristics including p53 deficiency [47, 48], AMPKα expression [34] and MYC status [49, 54].

Resistance of SK-N-BE(2c) cells to the 2-DG/metformin combination is likely to be explained by their genetic background, which includes p53 mutations [55] and amplification of the *MYCN* gene [56]. Amplification of the neuronal transcription factor MYCN induces malignant transformation of neuroblastoma cells and is used for disease stratification [57], and sensitivity of neuroblastoma cells to impaired polyamine metabolism has been demonstrated to correlate with extent of *MYCN* amplification [54]. We therefore extended our study to include CHLA-20 human neuroblastoma cells, which are non-*MYCN* amplified. Since CHLA-20 cells were unable to form clonogenic colonies, spheroid growth delay was used as a clinically relevant readout of radiosensitivity. In this cell line, dual treatment with 2-DG and metformin produced significantly greater radiosensitisation than either agent administered alone, supporting our findings in UVW/NAT cells and consistent with data derived from breast cancer [48, 50], prostate cancer [47] and paediatric glioma cells [49]. Our findings support a potential role for *MYCN* amplification in treatment resistance of neuroblastoma cells that merits further investigation.

Interrogation of the cellular metabolome by mass spectrometric analysis demonstrated significant disruption of the glycolytic pathway and the electron transport chain (ETC) following exposure to 2-DG and metformin as single agents. We further showed that the greatest metabolite imbalance was observed following combination of 2-DG and metformin treatments. Even though this imbalance was not further exacerbated by irradiation, it set the scene for enhanced radiosensitivity. Further investigations revealed that these large-scale metabolic imbalances ultimately culminated in reduced cellular energy production. These results were corroborated by real-time analysis of oxygen consumption rate using the Seahorse system, wherein triple combination therapy produced a substantial shift in bioenergetic profile from a highly energetic to a largely quiescent cellular phenotype. Additionally, we observed significantly reduced ATP production, glycolytic rate, and respiratory capacity following exposure to combination treatments. Similar analysis of biogenetic profiles proved useful during the characterisation of different histological subtypes of ovarian cancer, in which highly glycolytic cell subtypes exhibited greater susceptibility to 2-DG [58]. A key question is how do the metabolic imbalances detailed in this study relate to cell death following radiotherapy? Our data support a model in which (1) metabolic shifts from a highly energetic state to cellular quiescence prevent proliferation and (2) increased reliance on mitochondrial energy production following glycolytic inhibition increases production of reactive oxygen species (ROS), which enhances radiation-induced ROS production, thus increasing cytotoxicity. We hypothesise that inhibition of glycolysis by 2-DG causes a metabolic shift to oxidative phosphorylation (OXPHOS) in order to sustain ATP production, a compensatory mechanism that can be circumvented by adding metformin. Albeit by alternative methodologies, several other studies have also demonstrated reduced ATP content as a possible mechanism of additivity when 2-DG is combined with metformin [47, 49, 50].

Of course, the radiosensitising effect of 2-DG may not be solely due to inhibition of glycolysis. 2-DG is known to exert off-target affects as a mannose mimetic and can cause post-translational modifications such as N-linked glycosylation [59]. Indeed, N-linked glycosylation, in which an oligosaccharide is attached to a protein residue via its nitrogen atom, is a post-translational event analogous to poly(ADP-ribosyl)ation, whereby ADP-ribose moieties are attached to a target protein at the site of DNA damage. We have previously demonstrated the radiosensitising properties of the poly(ADP-ribose) polymerase-1 (PARP-1) inhibitors olaparib and rucaparib in neuroblastoma and glioma cells, where they act principally by preventing repair of radiation-induced damage [38]. Indeed, this radiation-induced poly-(ADP-ribosyl)ation which would consume pools of NAD^+^ and ADP, would occur synonymously with the ATP depletion following combined 2-DG/metformin treatment. Presumably, AMP supplies would be depleted in attempts to replenish ADP/ATP levels. This may be a possible explanation as to why we observed significant reductions in abundance of AMP, ADP, ATP and NAD^+^ following combination therapy.

In addition to its well-documented effects on glucose metabolism, we also demonstrated that 2-DG treatment had significant effects on purine and pyrimidine metabolism. Of note were the changes observed in ethanolamine phosphate and hypoxanthine, which are generated by carboxylation of serine and deamination of adenine, respectively. These metabolites were significantly increased by dual and triple combination therapies, but not by single agent treatments. Their increased abundance may reflect attempts to repair radiation-induced DNA damage, which require cells to increase de novo purine and pyrimidine synthesis, with input from serine and adenine, to synthesise the nucleotides required for DNA repair. Indeed, nucleotide metabolism has been implicated in oncogene-induced cellular senescence due to the roles of nucleotides in replication stress and the DNA damage response [60].

Flow cytometric analysis revealed that although 2-DG and metformin, as single agents, induced modest accumulation of cells in the G_2_/M phase of the cell cycle, maximum G_2_/M arrest was achieved by combining the two agents, as previously shown by others [47, 48]. Our data also demonstrated that this G_2_/M arrest could be further enhanced by combination with X-irradiation and, more importantly, that it persisted for at least 24 h after irradiation. This indicated that optimal scheduling of therapeutic modalities could maximise arrest in G_2_/M — the most radiosensitive phase of the cell cycle. Indeed, we went on to show that scheduling X-irradiation exposure 6 h before 2-DG/metformin treatment maximised G_2_/M arrest. However, this treatment schedule did not further enhance clonogenic cell kill compared to the other two schedules, which is why simultaneous combinations were used for the rest of the study. We predicted that the mechanism by which the 2-DG/metformin combination increased radiosensitivity entailed induction of apoptosis during prolonged G_2_/M accumulation of cells, as shown previously [47, 49, 50]. This hypothesis was supported by our Incucyte data which showed the frequency of Annexin V-stained UVW/NAT and CHLA-20 cells to be significantly enhanced following 2-DG/metformin treatment, compared to single agent administration.

Like most other in vitro studies investigating this drug combination [47–50], we observed cytotoxic activity of 2-DG and metformin only at higher drug concentrations than could be achieved clinically. In contrast, we observed potent radiosensitising effects of the combination with lower concentrations (1 mM). Clinically, the recommended dose of 2-DG is 63 mg/kg/day (75 μM^1^), which achieved maximum plasma concentrations in the region of 116 μg/ml (0.7 μM) [23], although higher doses may be tolerated in combination with radiation therapy. And while metformin is not usually given to diabetic patients at doses higher than 1500–2000 mg/day, which deliver plasma concentrations of 22-29 μM^1^ [27], it should be noted that shorter treatment regimens would be required in combination with radiotherapy, so it may be feasible to treat neuroblastoma patients with higher daily doses. Since both drugs are already clinically approved, exhibit favourable toxicity profiles and, as far as the authors are aware, have no documented maximum tolerated dose to date, we consider them to be promising therapeutic agents in combination with radiation therapy in paediatric patients.

## Conclusions

In summary, we show for the first time that the glycolytic inhibitor 2-DG enhances the radiosensitivity of human neuroblastoma and glioma cells in vitro. The radiosensitising effects of 2-DG were greatly enhanced by combination with the antidiabetic biguanide, metformin. Metabolomic analysis revealed this combination to elicit severe disruption of key glycolytic and mitochondrial metabolites, causing significant reductions in ATP generation. These results were supported by cellular bioenergetic profiling. Combination treatment induced G_2_/M arrest that persisted for at least 24 h post-irradiation, promoting apoptotic cell death in a significant proportion of cells. Our findings suggest that targeting cellular metabolism has potential to improve clinical outcomes by overcoming radioresistance in children with high-risk neuroblastoma.

## Supplementary Information

**Additional file 1: Supplementary Figure S1**. The effect of 2-DG and metformin on cell survival. **Supplementary Figure S2**. The radiosensitising effect of 2-DG and metformin as single agents. **Supplementary Figure S3**. The effect of combination treatment on glycolytic and mitochondrial metabolites. **Supplementary Figure S4**. Effects of 2-DG treatment on purine metabolism. **Supplementary Figure S5**. Effects of 2-DG treatment on pyrimidine metabolism. **Supplementary Figure S6**. The effect of combination treatment on mitochondrial respiration, glycolysis and cellular energy production. **Supplementary Figure S7**. The effect of combination treatment on mitochondrial respiration, glycolysis and cellular energy production. **Supplementary Figure S8**. The effect of 2-DG and metformin on the cell cycle. **Supplementary Figure S9**. The effect of combination treatment on apoptotic frequency.

## Data Availability

The datasets used and/or analysed during the current study are available from the corresponding author on reasonable request.

## Abbreviations

2DG: 2-Deoxyglucose
AUC: Area under the curve
DEF_50_: Dose enhancement factor observed at the 50% kill level
DMEM: Dulbecco’s modified Eagle medium
ECAR: Extracellular acidification rate
HILIC: Hydrophilic interaction liquid chromatography
IC_50_: 50% Inhibitory concentration
IR: Irradiated/irradiation
MIBG: Meta-iodobenzylguanidine
NAT: Noradrenaline transporter
OCR: Oxygen consumption rate
OXPHOS: Oxidative phosphorylation
PARP-1: Poly(ADP-ribose) polymerase-1
PiMP: Polyomics integrated Metabolomics Pipeline
SSP: Staurosporine
TCA: Tricarboxylic acid

## Footnotes

1 Assuming 80 kg average weight and 5.15 L average circulating blood volume.
