# Supplementary material for: Inhibition of glycolysis and mitochondrial respiration promotes radiosensitisation of neuroblastoma and glioma cells
Source: Cancer Metab. 2021 May 19;9:24. doi: 10.1186/s40170-021-00258-5 (PMC8136224; doi:10.1186/s40170-021-00258-5)
Supplement: Supplementary file 1 — Additional file 1: Supplementary Figure S1. The effect of 2-DG and metformin on cell survival. Supplementary Figure S2. The radiosensitising effect of 2-DG and metformin as single agents. Supplementary Figure S3. The effect of combination treatment on glycolytic and mitochondrial metabolites. Supplementary Figure S4. Effects of 2-DG treatment on purine metabolism. Supplementary Figure S5. Effects of 2-DG treatment on pyrimidine metabolism. Supplementary Figure S6. The effect of combination treatment on mitochondrial respiration, glycolysis and cellular energy production. Supplementary Figure S7. The effect of combination treatment on mitochondrial respiration, glycolysis and cellular energy production. Supplementary Figure S8. The effect of 2-DG and metformin on the cell cycle. Supplementary Figure S9. The effect of combination treatment on apoptotic frequency. [file 40170_2021_258_MOESM1_ESM.docx]

**Supplementary Information**

**
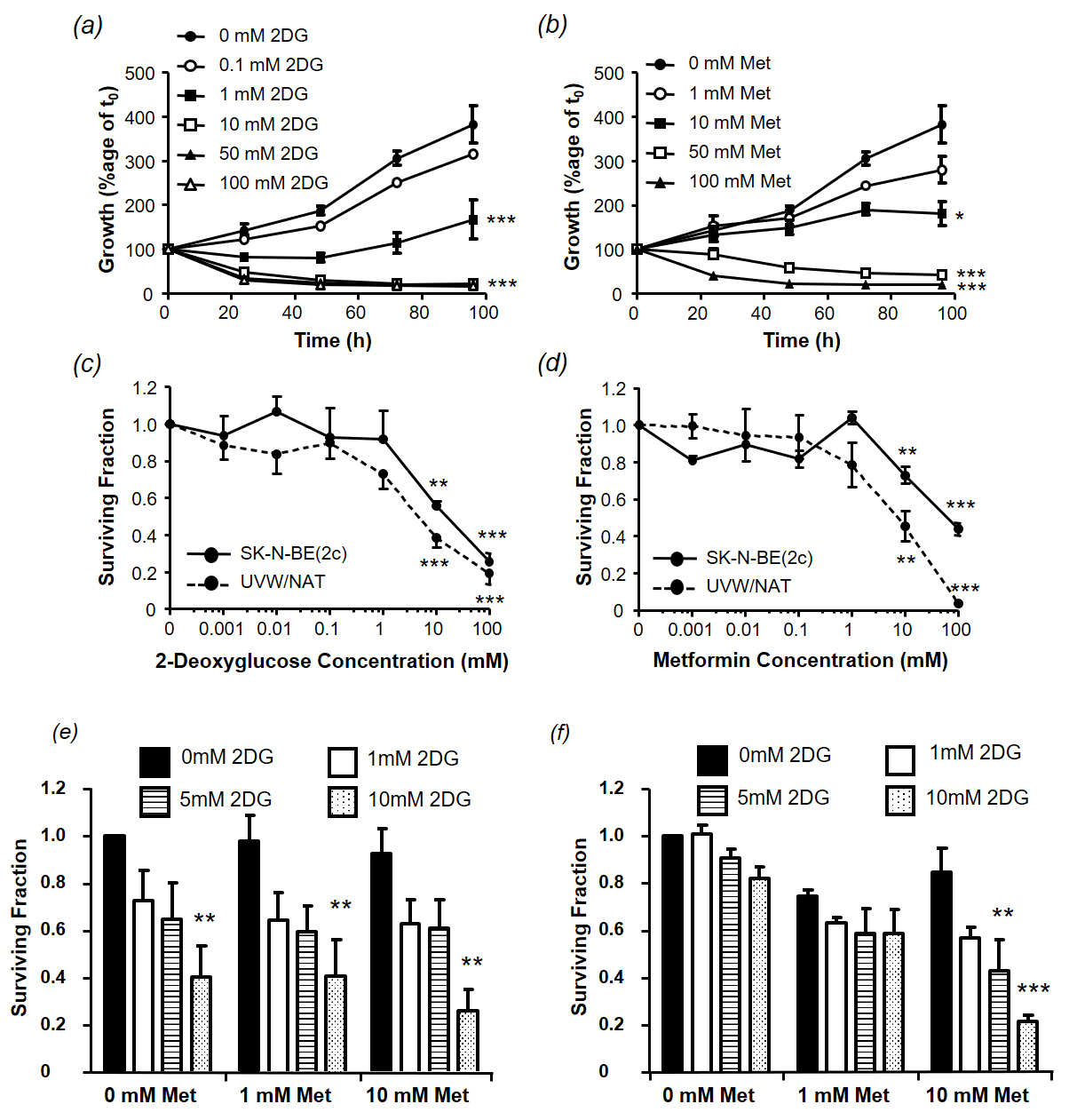
**

**Supplementary Figure S1: The effect of 2-DG and metformin on cell survival**. SK-N-BE(2c) cells were treated with increasing concentrations of (a) 2-DG or (b) metformin for 98 h. MTT assays were performed every 24 h to assess cell proliferation. The area under the curve (AUC) was calculated for each drug treatment and compared to that of control cells using one-way ANOVA with Bonferroni correction (*p<0.05, ***p<0.001). The effect of 24 h (c) 2-deoxyglucose and (d) metformin treatment on the clonogenic survival of SK-N-BE(2c) and UVW/NAT cells. The clonogenic survival of (e) UVW/NAT and (f) SK-N-BE(2c) (n=4) cells following 24 h treatment with 2-DG (1, 5, 10 mM) or metformin (1 or 10 mM), as single agents or in simultaneous combination. **p<0.01, ***p<0.001 compared to untreated control cells. Data are the mean ± SEM from at least 3 independent experiments, unless otherwise stated.


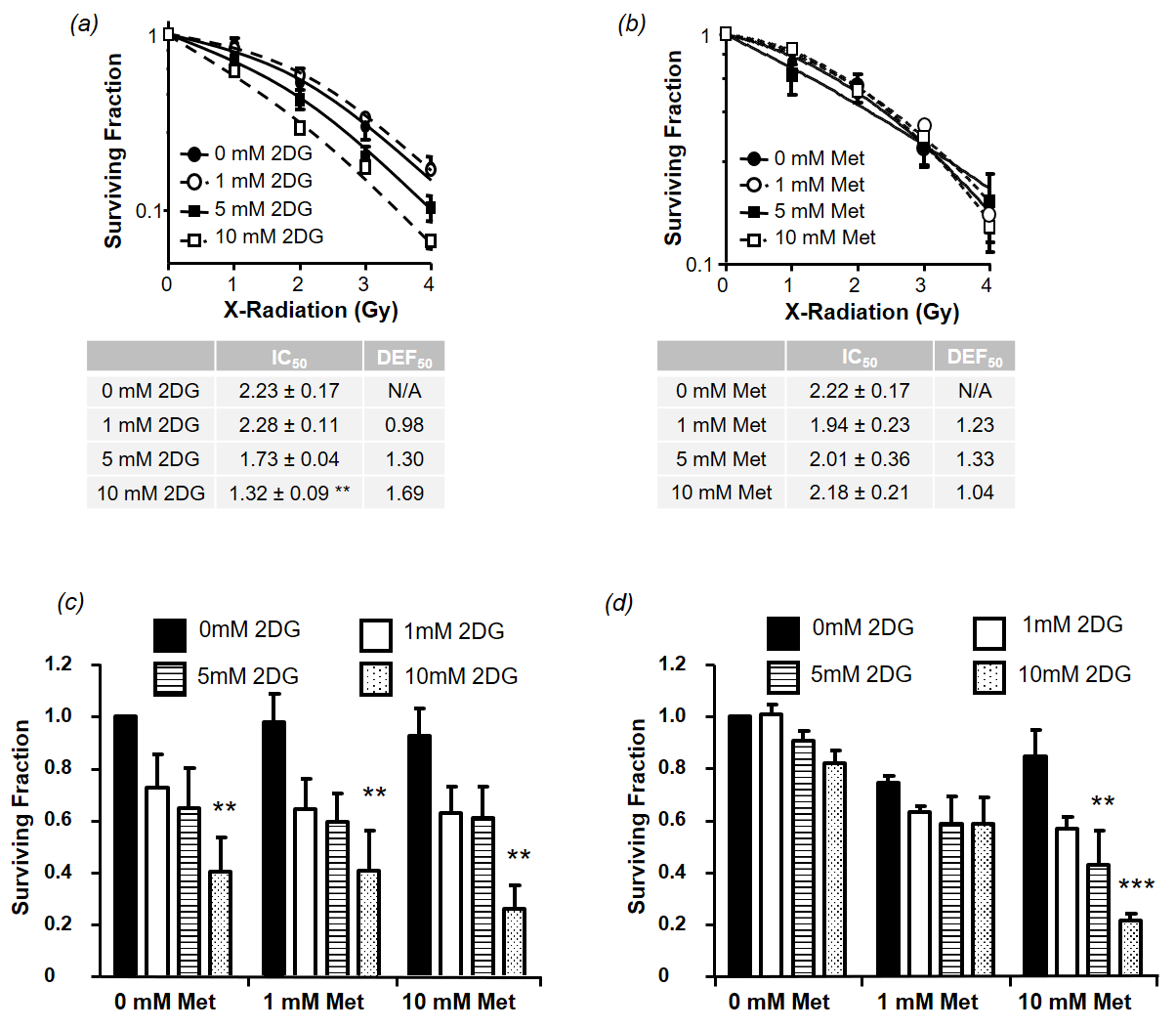


**Supplementary Figure S2: The radiosensitising effect of 2-DG and metformin as single agents**. The clonogenic survival of SK-N-BE(2c) cells following 24 h treatment with (a) 1, 5 or 10 mM 2-DG or (b) 1, 5 or 10 mM metformin in the presence 1-4 Gy X-radiation. The 50% inhibitory concentration (IC_50_) and the dose enhancement factor observed at the 50% kill level (DEF_50_) are shown. The combined effect of 2-DG and metformin on the clonogenic survival of (c) SK-N-BE(2c) cells and (d) UVW/NAT cells. *p<0.05, **p<0.01 compared to untreated control cells. All data are mean ± SEM from 3 experimental repeats.

**
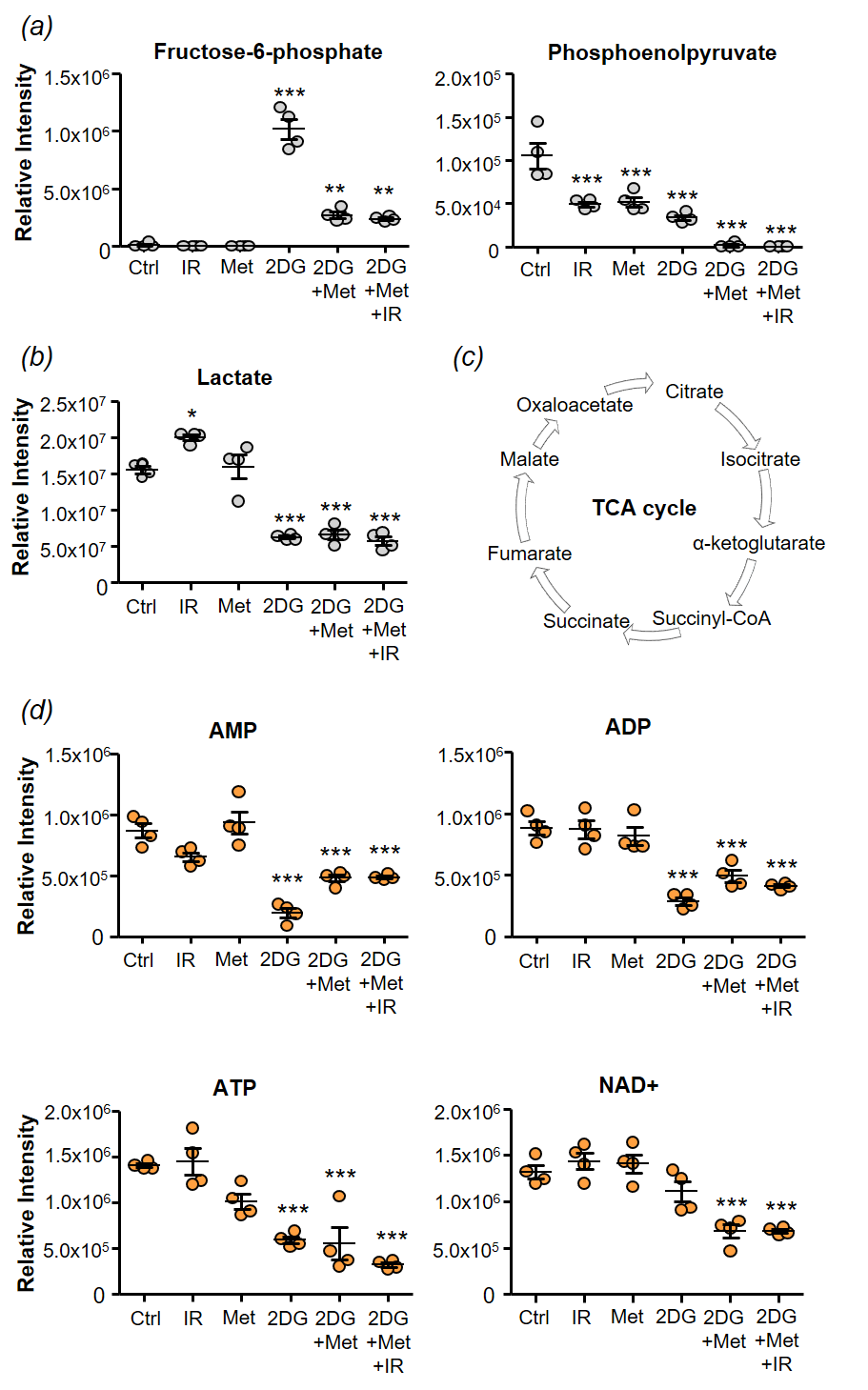
**

**Supplementary Figure S3: The effect of combination treatment on glycolytic and mitochondrial metabolites.** Intracellular relative intensity of (a) glycolytic intermediates, (b) lactate, an anaerobic glycolysis waste product, and (d) energy metabolites following mass spectrometry and 24 h treatment with 3 Gy X-radiation, 1 mM metformin, or 2.5 mM 2-DG as single agents or in triple combination. (c) Schematic representation of the mitochondrial tricarboxylic acid (TCA) cycle. Data are means ± SD, n=4. * p< 0.05, ** p< 0.01, *** p<0.001 following a one-way ANOVA with Bonferroni correction.

**
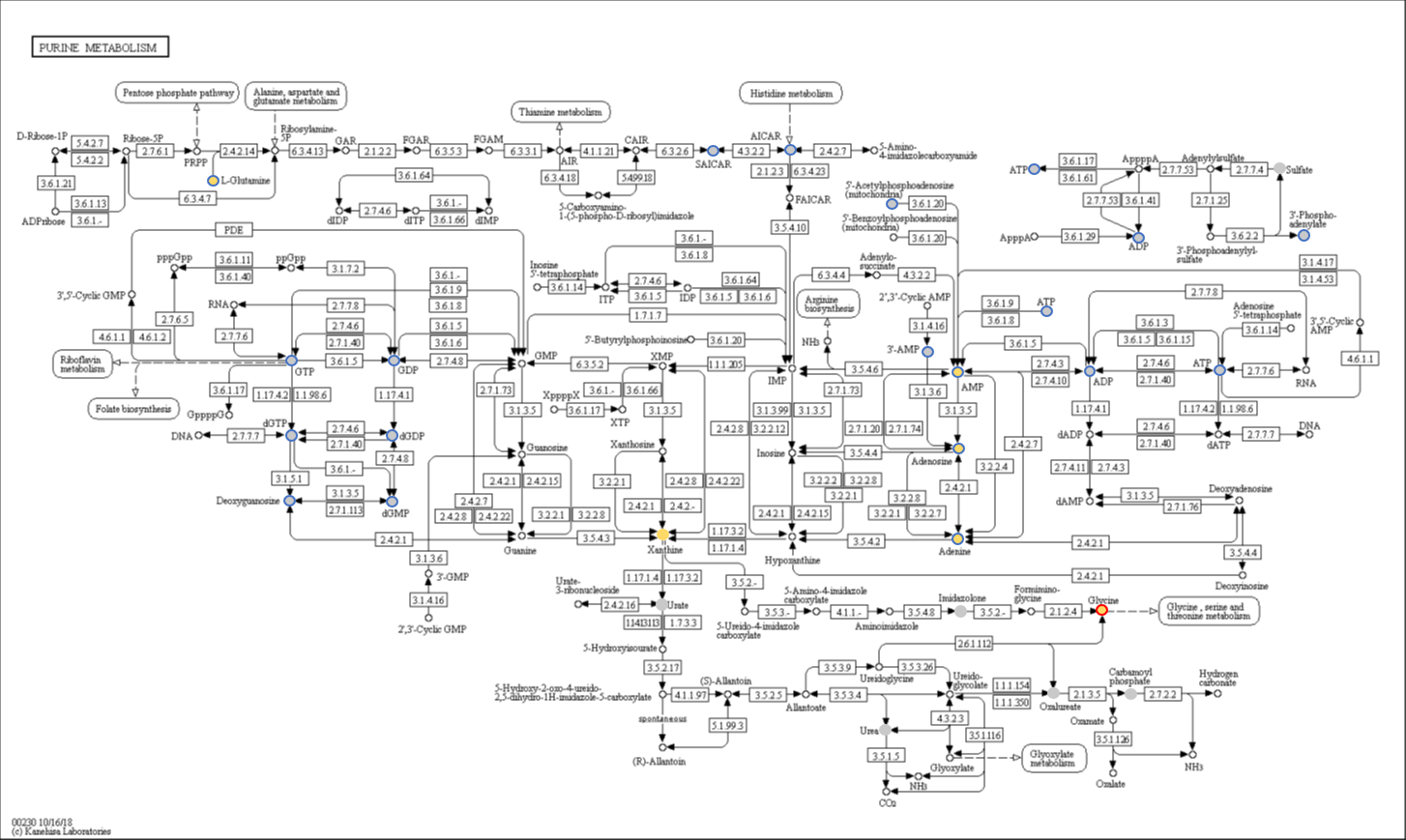
**

**Supplementary Figure S4: Effects of 2-DG treatment on purine metabolism.** Map of metabolism extracted from KEGG (www.genome.jp/kegg). Detected metabolites (circles) are coloured grey (annotated) or yellow (annotated and matches the retention time of an authentic standard). Metabolite outlines are coloured red (increase in 2-DG compared to untreated) or blue (decrease in 2-DG compared to untreated).

**
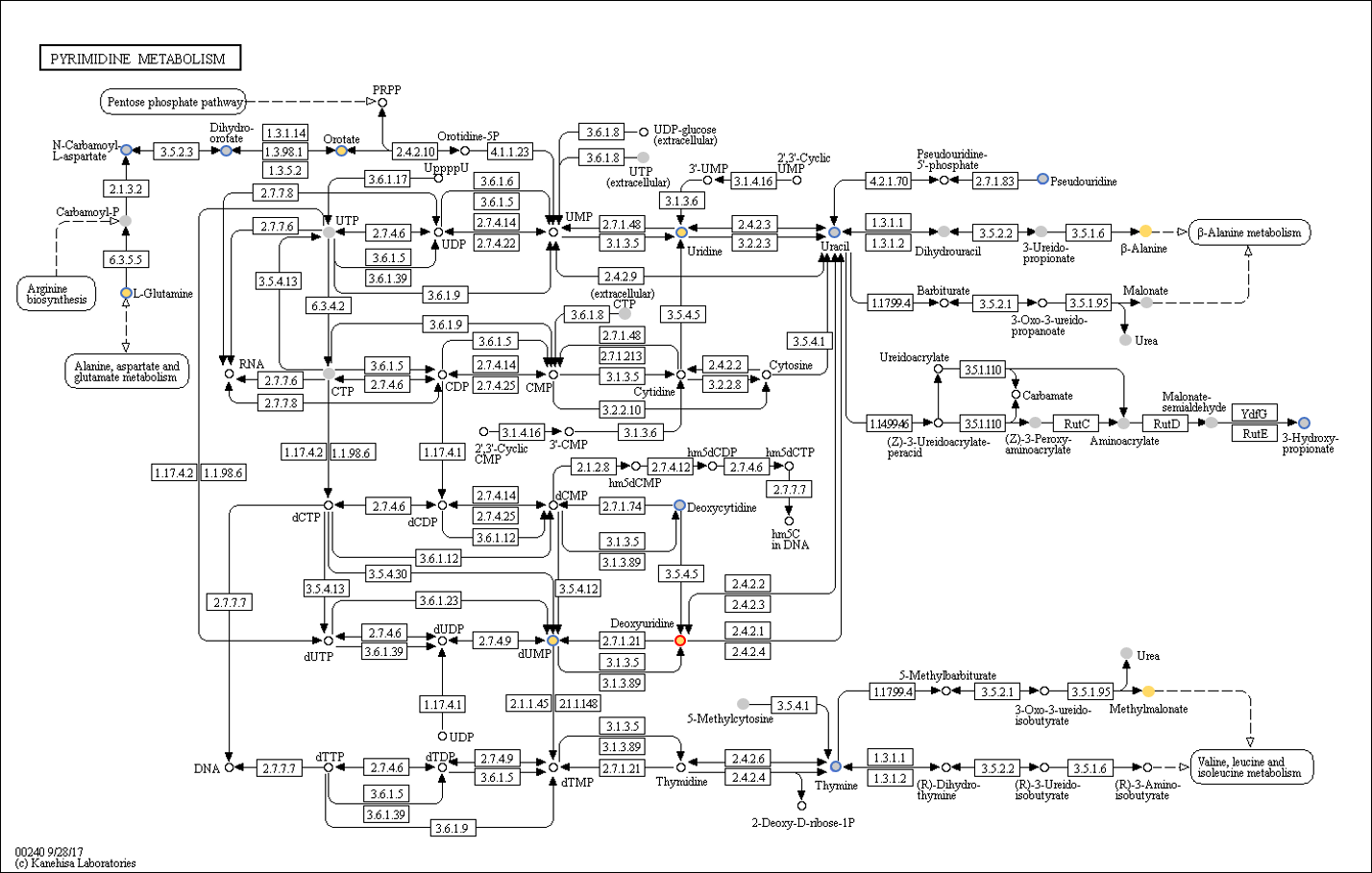
**

**Supplementary Figure S5: Effects of 2-DG treatment on pyrimidine metabolism.** Map of metabolism extracted from KEGG (www.genome.jp/kegg). Detected metabolites (circles) are coloured grey (annotated) or yellow (annotated and matches the retention time of an authentic standard). Metabolite outlines are coloured red (increase in 2-DG compared to untreated) or blue (decrease in 2-DG compared to untreated).

**
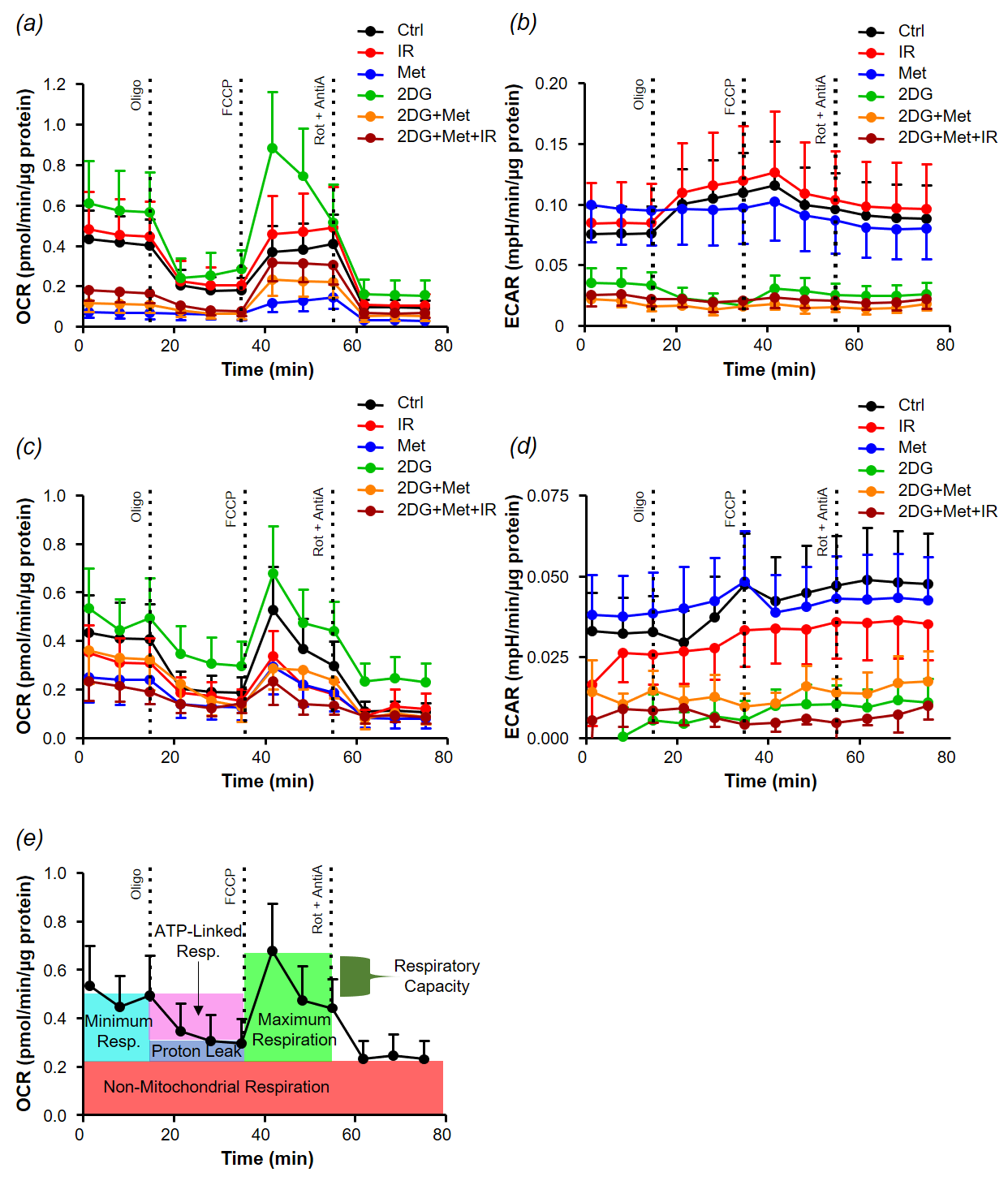
**

**Supplementary Figure S6: The effect of combination treatment on mitochondrial respiration, glycolysis and cellular energy production.** UVW/NAT cells (a, b) and CHLA20 cells (c, d) were treated with 3 Gy X-radiation, 1 mM metformin, or 5 mM 2-DG as single agents or in combination for 24 h. Oxygen consumption rate (OCR) (a, c) and extracellular acidification rate (ECAR) (b, d) of live cells were then measured using a Seahorse XFe96 Analyser. Data are means ± SEM, n=4 (each experiment performed with 6 replicates). The traces obtained were then used to calculate minimal and maximal respiratory capacity, ATP production and glycolytic rate (non-mitochondrial respiration) as indicated in (e).

**
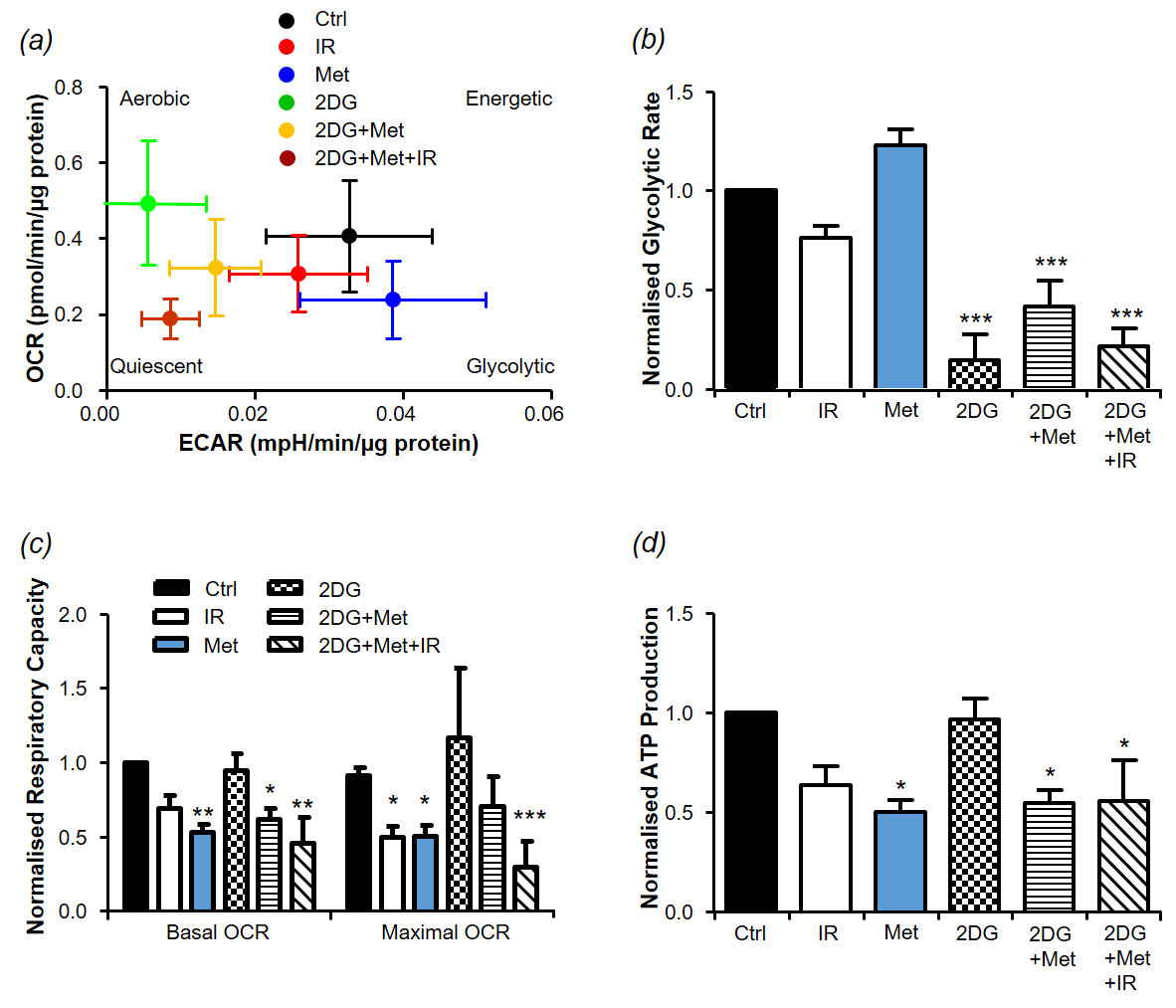
**

**Supplementary Figure S7: The effect of combination treatment on mitochondrial respiration, glycolysis and cellular energy production.** CHLA20 cells were treated with 3 Gy X-radiation, 1 mM metformin, or 5 mM 2-DG as single agents or in combination for 24 h. Oxygen consumption rate (OCR) and extracellular acidification rate (ECAR) of live cells were then measured using a Seahorse XFe96 Analyser, the shift in cell energy phenotype following treatment is shown (a). The traces obtained (Supplementary Figure 6) were then used to calculate glycolytic rate (b), minimal and maximal respiratory capacity (c) and ATP production (d).

**
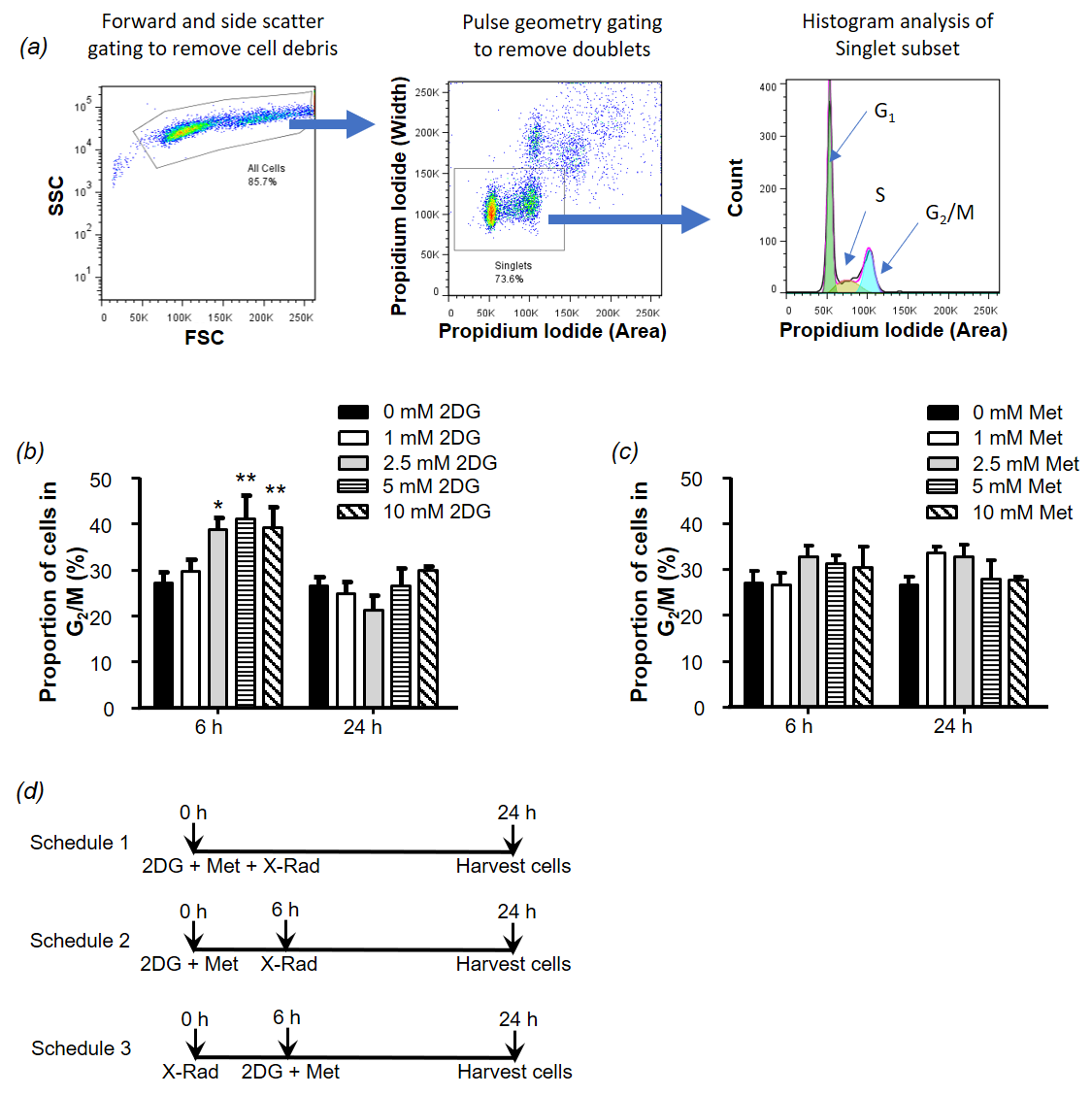
**

**Supplementary Figure S8: The effect of 2-DG and metformin on the cell cycle.** Our gating strategy for analysing cells using flow cytometry is shown (a), whereby viable cell populations were first selected using forward and side scatter gating, followed by doublet discrimination using pulse geometry gating. The remaining singlet cell population was visualised as a histogram and the Dean-Jett-Fox algorithm in FlowJo was used to determine the proportion of cells in G_1_ (green peak), S (yellow peak) and G_2_/M phase (blue peak) of the cell cycle. UVW/NAT cells were treated with (b) 1-10 mM 2-DG or (c) 1-10 mM metformin as single agents for 6 or 24 h. Data show flow cytometric analysis of cell cycle distribution following propidium iodide staining. (d) Administration of 3 Gy X-radiation, 1 mM metformin, or 2.5 mM 2-DG as three different schedules. Data presented are means ± SEM from 3 independent experiments * p<0.05, **p<0.01 compared to untreated control cells.


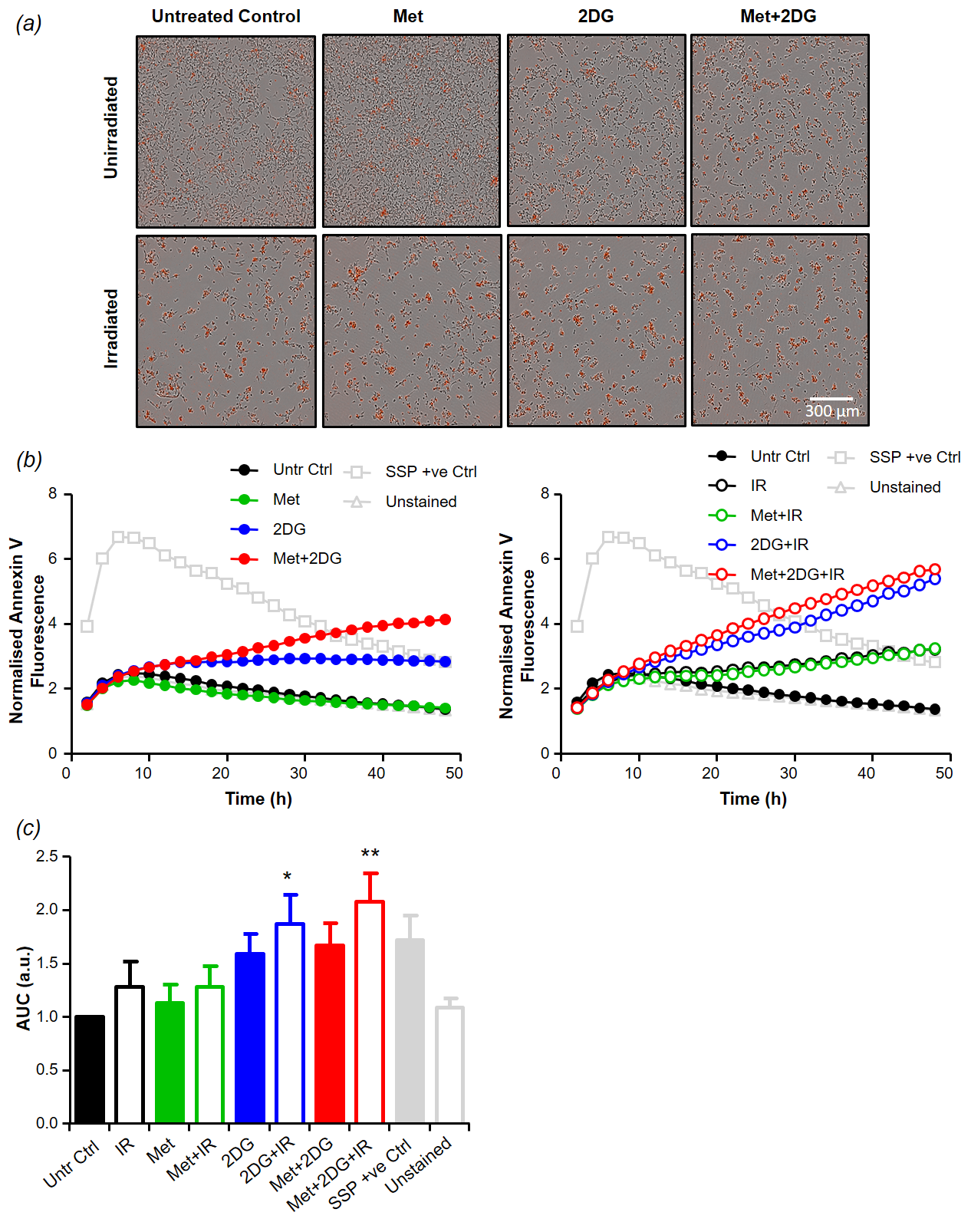


**Supplementary Figure S9: The effect of combination treatment on apoptotic frequency.** CHLA20 cells were treated with 2 Gy X-radiation, 1 mM metformin, or 1 mM 2-DG as single agents or in combination for 48 h and were imaged every hour using the IncuCyte Zoom imaging system. Representative images taken after 48 h treatment exposure are shown (a). Apoptotic frequency was determined following Annexin V staining. Annexin V positive cells (displaying red fluorescence) were normalised to the total number of cells present (b) and the area under the curve (AUC) was calculated (c) to allow comparisons between each condition and the treatment controls (untreated control (Untr Ctrl), unstained negative control (unstained) and staurosporine positive control (SSP +ve)). Data are means ± SEM from 3 separate experiments, each performed in sextuplet wells. *p<0.05; **p<0.01 compared to untreated control cells (one-way ANOVA).
